# Cognitive change is more positively associated with an active lifestyle than with training interventions in older adults at risk of dementia: a controlled interventional clinical trial

**DOI:** 10.1186/s12888-016-1018-z

**Published:** 2016-09-08

**Authors:** Olivia C. Küster, Patrick Fissler, Daria Laptinskaya, Franka Thurm, Andrea Scharpf, Alexander Woll, Stephan Kolassa, Arthur F. Kramer, Thomas Elbert, Christine A. F. von Arnim, Iris-Tatjana Kolassa

**Affiliations:** 1 Clinical and Biological Psychology, Institute of Psychology and Education, Ulm University, 89081 Ulm, Germany; 2 Department of Psychology, TU Dresden, Dresden, Germany; 3 Institute of Sports and Sports Science, Karlsruhe Institute of Technology, Karlsruhe, Germany; 4 SAP Switzerland AG, Tägerwilen, Switzerland; 5 Beckman Institute for Advanced Science and Technology, University of Illinois Champaign-Urbana, Champaign, IL USA; 6 Department of Psychology, University of Konstanz, Konstanz, Germany; 7 Department of Neurology, Ulm University, Ulm, Germany

**Keywords:** Mild cognitive impairment, Alzheimer’s disease, Activity, Active lifestyle, Cognition, Training, Exercise

## Abstract

**Background:**

While observational studies show that an active lifestyle including cognitive, physical, and social activities is associated with a reduced risk of cognitive decline and dementia, experimental evidence from corresponding training interventions is more inconsistent with less pronounced effects. The aim of this study was to evaluate and compare training- and lifestyle-related changes in cognition. This is the first study investigating these associations within the same time period and sample.

**Methods:**

Fifty-four older adults at risk of dementia were assigned to 10 weeks of physical training, cognitive training, or a matched wait-list control condition. Lifestyle was operationalized as the variety of self-reported cognitive, physical, and social activities before study participation. Cognitive performance was assessed with an extensive test battery prior to and after the intervention period as well as at a 3-month follow-up. Composite cognition measures were obtained by means of a principal component analysis. Training- and lifestyle-related changes in cognition were analyzed using linear mixed effects models. The strength of their association was compared with paired *t*-tests.

**Results:**

Neither training intervention improved global cognition in comparison to the control group (*p* = .08). In contrast, self-reported lifestyle was positively associated with benefits in global cognition (*p* < .001) and specifically in memory (*p* < .001). Moreover, the association of an active lifestyle with cognitive change was significantly stronger than the benefits of the training interventions with respect to global cognition (*p*s < .001) and memory (*p*s < .001).

**Conclusions:**

The associations of an active lifestyle with cognitive change over time in a dementia risk group were stronger than the effects of short-term, specific training interventions. An active lifestyle may differ from training interventions in dosage and variety of activities as well as intrinsic motivation and enjoyment. These factors might be crucial for designing novel interventions, which are more efficient than currently available training interventions.

**Trial registration:**

ClinicalTrials.gov Identifier NCT01061489. Registered February 2, 2010.

**Electronic supplementary material:**

The online version of this article (doi:10.1186/s12888-016-1018-z) contains supplementary material, which is available to authorized users.

## Background

With increasing life expectancy, prevention and treatment of cognitive decline and dementia becomes a major topic in the debate on successful aging. In observational studies, an active lifestyle has been identified as a protective factor against cognitive decline and dementia [1, 2]. Individuals who reported high levels of physical [3] or cognitive activity [4] had a substantially reduced risk of cognitive impairment of 38 to 50 % in comparison to sedentary individuals. Interestingly, the accumulated leisure time spent with activities per week seems to be less important than the number of different physical [5, 6] or cognitive activities [7]. Furthermore, engagement in multiple activity domains (social, cognitive, physical) seems to be particularly beneficial to prevent cognitive decline [8, 9].

However, experimental trials with physical or cognitive training interventions are needed to make inferences about the causality of effects. In comparison to the results of the observational studies, interventional trials yielded smaller and more inconsistent effects: With regard to physical training, some studies reported cognitive improvements after training interventions in healthy older adults [10, 11], adults with elevated risk of Alzheimer’s disease [12, 13] or older adults with dementia [14], while other studies failed to find beneficial effects [15–17]. A number of meta-analyses over the past decade have helped to clarify the literature that has examined physical training effects on measures of cognition [18–22]. In general, these meta-analyses have found modest effect sizes for this relationship. For instance, Smith et al. [20] reported small effects sizes on different cognitive domains (Hedges *g* between 0.12 and 0.16). As to cognitive training, beneficial effects on cognition have been reported [23]. However, the applied training tasks were often quite similar to the outcome measures in the studies, and training effects were restricted to the trained domain [23, 24]. There is an intensive debate on the extent to which improvements through training generalize to broader cognitive constructs, and especially to everyday cognitive functioning [25–27]. Lately, a novel cognitive training approach was developed, based on principles of neuroplasticity [28]. This approach focusses on the training of auditory discrimination abilities and working memory [29, 30]. Mahncke and colleagues could demonstrate that verbal memory performance increased in healthy older adults after 8 to 10 weeks of training with this program [31, 32]. However, in participants at risk of dementia, this training program yielded inconsistent results [16, 33, 34].

In summary, there are beneficial effects of training interventions on cognition, although they appear to be less pronounced than associations of activity with cognitive change in observational studies. The gap between promising observational evidence, demonstrating substantial cognitive benefits of physical and cognitive activities, and more equivocal results from interventions may result from differing characteristics of the investigated activities in observational and interventional studies, for example, differences in duration, variety, multimodality, or intrinsic motivation and enjoyment of the activities. The studies are however difficult to compare, as the observation periods are entirely different. Prospective studies often apply a time frame of several years, while interventions in the experimental studies rarely last longer than several weeks or months. This is the first study, which directly compares training- and lifestyle-related changes in cognition within the same sample and time period.

The first objective of this study was to evaluate intervention effects on cognition, while considering lifestyle-related changes in cognition. We applied a cognitive and a physical training program in a sample of older adults with memory complaints. To date, there is only a small number of studies with inconsistent results in this population at risk. The second, exploratory aim was to compare the training- and lifestyle-related changes in cognition. Lifestyle was defined in terms of the number of self-reported activities in the month before study participation. Thus, the focus is laid on the variety of activities, rather than their intensity or dosage. To our knowledge, this is the first study which compares training- and lifestyle-related changes in cognition within the same set of participants and the same time period.

## Methods

### Participants

**Fig. 1:**
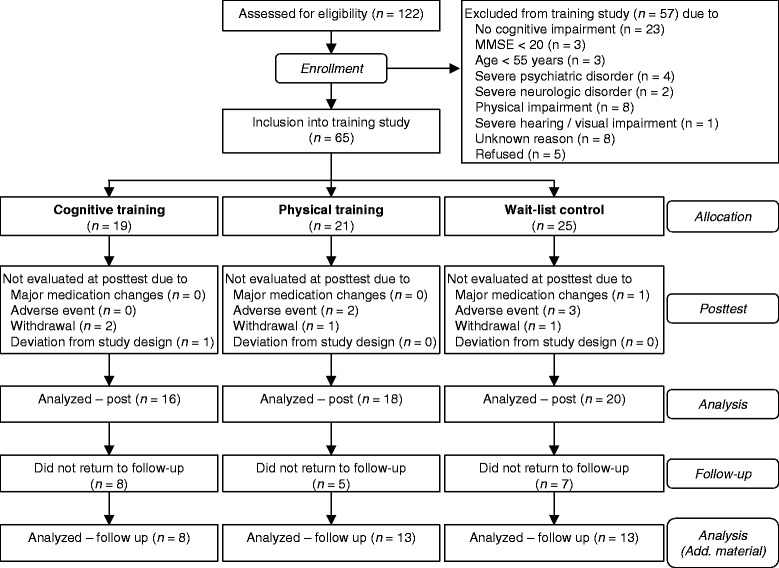
Flow of participants from screening to completion of the follow-up. Results regarding the follow-up are included in the Additional file 1

The study adheres to CONSORT guidelines. The study was conducted between 2009 and 2013 at two study sites in Germany, the University of Konstanz and the University of Ulm. Subjects were recruited in the memory clinics of the University Hospital Ulm and of the Reichenau Psychiatry Center in Konstanz and via newspaper articles, flyers, and informative meetings at both study sites. One hundred twenty-two older adults were screened for eligibility. We included individuals aged 55 years or older with subjective memory complaints and objective or clinically apparent memory impairment, vision and hearing adjusted to normal, and fluency in German language. Exclusion criteria were a history of severe psychiatric or neurologic disorders, a moderate or severe stage of dementia (Mini-Mental State Examination [MMSE] < 20^1^), changes in antidementive or antidepressive medication within 3 months prior to study initiation, or physical conditions which would prevent a participation in the physical training program (see Fig. 1). Sixty-five participants^2^ were enrolled into the intervention study. Due to dropouts, the data of 54 subjects were analyzed with a mean age of 71.4 years (*SD* = 5.9 years, range 60–88 years), of whom 16 had been allocated to the cognitive training group (CT), 18 to the physical training group (PT), and 20 to the wait-list control group (WLC). The three groups (CT, PT, WLC) did not differ significantly in sociodemographic variables, medication, cognitive performance, or baseline lifestyle activity (see Table 1). Lifestyle was not significantly correlated to cognition at baseline (see Table 2).

**Table 1:** Demographic and lifestyle characteristics and baseline cognitive performance within the three intervention groups

Variable	CT (*n* = 16)	PT (*n* = 18)	WLC (*n* = 20)	Statistic	*p*
Age: *M* (*SD*)	70.2 (5.8)	73.7 (6.2)	70.3 (5.5)	*F* (2,51) = 2.11	0.13
Gender: male / female	8 / 8	6 / 12	10 / 10	*χ* ^2^(2) = 1.35	0.51
Education in years: *M* (*SD*)	13.3 (4.0)	14.2 (3.0)	15.2 (3.7)	*F*(2,51) = 1.18	0.32
MMSE: *M* (*SD*)	27.8 (2.6)	27.8 (1.7)	28.2 (2.2)	*F*(2,51) = 0.14	0.87
WST z-score: *M* (*SD*)	0.64 (0.57)	0.69 (0.96)	1.01 (0.92)	*F*(2,51) = 1.07	0.35
Global cognition: *M* (*SD*)	0.08 (0.64)	0.04 (0.62)	-0.10 (0.82)	*F*(2,51) = 0.33	0.72
Memory: *M* (*SD*)	-0.02 (0.83)	0.16 (0.67)	-0.11 (0.98)	*F*(2,51) = 0.48	0.62
Attention / executive functions: *M* (*SD*)	0.19 (0.64)	-0.08 (0.75)	-0.08 (0.78)	*F*(2,51) = 0.75	0.48
Number of reported activities: *M* (*SD*)	8.4 (3.4)	8.7 (2.5)	9.3 (2.5)	*F*(2,49) = 0.39	0.68
Variety of activities^a^: *M* (*SD*)	0.27 (0.13)	0.28 (0.09)	0.30 (0.09)	*F*(2,49) = 0.60	0.55
Antidementive medication: no / yes	11 / 5	17 / 1	18 / 2	*χ* ^2^(4) = 5.80	0.21
Antidepressants: no / yes	15 / 1	18 / 0	19 / 1	*χ* ^2^(2) = 1.08	0.58

Depicted are means (*M*) and standard deviations (*SD*) in parentheses

*CT* Cognitive training group, *PT* Physical training group, *WLC* Wait-list control group, *MMSE* Mini-Mental State Examination, *WST* German vocabulary test as a measure for premorbid intelligence, *dementia* probable dementia

^a^Average score of physical, cognitive, and social activities domain scores, which represent the proportion of performed activities in relation to the possible number of activities in the respective domain

**Table 2:** Associations of lifestyle with demographic variables and cognition at baseline

Variable	*r*	*p*
Age	-0.18	0.21
Education in years	0.48	<0.001
MMSE	0.22	0.13
WST z-score	0.40	<0.001
Global cognition	0.23	0.11
Memory	0.17	0.23
Attention / executive functions	0.24	0.08

*MMSE* Mini-Mental State Examination, *WST* German vocabulary test as a measure for premorbid intelligence

### Procedure

Participants were screened for eligibility and socio-demographic data were assessed. Cognitive tests were performed with eligible subjects at a pre-test within one or two appointments. In addition, lifestyle was assessed in all participants at this time-point. Subsequently, the participants were allocated to the three groups (CT, PT, and WLC). Due to logistic issues, a randomized allocation to the groups was not feasible. To avoid a selection bias, the groups were matched on age, education, gender and cognitive status (MMSE). The PT intervention, carried out in small groups, required five to ten participants at a time when starting a new training group. At these time-points, all participants who had currently finished the screening and were included in the study were allocated to the PT group until the required number of participants was reached. In the following time periods the participants were allocated to the CT and WLC group using a minimization approach, in order to minimize differences in age, gender, education and cognitive status (MMSE) between the groups.

**Fig. 2:**
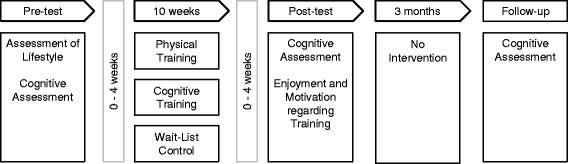
Study procedure. Participants underwent a pre-test, including the assessment of lifestyle and of cognitive measures. Participants were then assigned to one of three training groups, which started up to 4 weeks after the pre-test. Up to 4 weeks after the last training session, the post-test was arranged. A follow-up was conducted after further 3 months

The training sessions or waiting period started 1 to 4 weeks after the pre-test and lasted 10 weeks (see Fig. 2). Training duration was in accordance with typical durations of the applied cognitive training program [31, 32]. After the last training session the post-test was arranged. Time intervals between pre- and post-tests were similar in the WLC group. A follow-up assessment was carried out after another 3 months. Post-test and follow-up included the same cognitive tests as the pre-test plus a short questionnaire on the feasibility of the training programs. The investigators who conducted the neuropsychological assessment were blinded to the subjects’ group assignments. This was not always maintained due to participant disclosure.

### Training interventions

#### Cognitive training

Participants performed 1 h training sessions five times per week for 10 weeks. Apart from one to two guided sessions in the beginning of the study, the training was performed at the participants’ homes individually. Every other week the participants were contacted via telephone to ensure performance and compliance. In some cases, family members of the participants were additionally instructed to supervise the training sessions at home. The computer-based training program was developed by the Posit Science Corporation (San Francisco, CA) and adapted and translated into German in cooperation with Posit Science. The training consisted of six different tasks which target the auditory discrimination of frequencies and syllables as well as working memory processes (for details see [32]). One of the original tasks (“listen and do”) was substituted by a task which targeted the frequency discrimination of sounds (“frequency discrimination”), as a translation of the original task into German would have been too complex. The training was programmed in a way that some of the tasks were executed more often than others and that the order of the tasks varied in each session. Within each task the difficulty of the auditory and working-memory elements was adapted on the basis of the participant’s performance. Correct answers were reinforced by specific sounds and the uncovering of a picture. Performance in the training tasks was assessed in each session. To evaluate improvements within the training, the scores of the third session and the last session were used for each of the four most frequently executed tasks (“high or low”, “tell us apart”, “sound replay”, and “match it”), as measures of beginning and final training performance, respectively (see Additional file 1).

#### Physical training

The PT was carried out in groups of five to ten participants. The groups attended 1 h training sessions twice a week for 10 weeks. In addition, homework sessions of around 20 min were completed three times a week at home. Homework sessions were documented by the participants and regularly checked by the instructors. We aimed to provide a program that can be carried out by older adults (without major walking disabilities) at home and that does not require much additional equipment or medical check-ups. The training program was therefore adapted from a program which previously yielded small, but positive effects in frail nursing home residents with dementia [14]. Besides endurance training, it also included coordination, balance, flexibility, and strengthening elements in order to keep participants motivated during the intervention. In each session these elements were integrated into an imaginary journey. The difficulty of the physical training was adapted individually by two instructors.

#### Wait-list control

Participants of the WLC group did not receive any intervention but were asked to continue their daily routine as usual and were offered to take part in one of the training programs after their study participation.

### Assessment of lifestyle

Physical, cognitive, and social activity are major protective lifestyle factors of dementia [35]. We thus operationalized lifestyle in this study by the amount of activity performed before study participation. By this means, the lifestyle measure and the training procedures were comparable in their nature, as both focused on (physical and cognitive) activity.

The Community Healthy Activities Model Program for Seniors Physical Activity Questionnaire for Older Adults [36] was used to assess lifestyle in all participants at pre-test. The questionnaire assesses frequency and duration of 40 different physical, cognitive, and social activities of a typical week within the previous 4 weeks. The questionnaire is valid for measuring physical activity [37], but also assesses a large number of social and cognitive activities. The activities were categorized into physical, cognitive, and social activity domains by three of the authors and an independent sample of older adults, with comparable results (see Additional file 1). Lifestyle was defined as the variety of reported activities. A domain score for each activity domain was built, reflecting the percentage of performed, domain-specific activities in relation to the possible number of activities in this domain. The three domain scores were averaged to one score, in order to represent the overall variety of activities, as the lifestyle measure.

### Cognitive assessment

A wide set of cognitive functions sensitive to age-related cognitive decline and dementia with different item-difficulty was assessed. Participants completed German versions of the MMSE [38], the Alzheimer’s Disease Assessment Scale – cognitive subscale [39], the test battery of the Consortium to Establish a Registry for Alzheimer’s Disease (without word list encoding, recall, and recognition) [40], the subtests digit span and digit-symbol-coding of the Wechsler Adult Intelligence Scale [41], and the working-memory subtest of the Everyday Cognition Battery [42]. In addition, an adapted German version of the California Verbal Learning Test (J. Ilmberger: Münchner Verbaler Gedächtnistest MVGT [ Munich verbal memory test], unpublished) was conducted. The Geriatric Depression Scale-15 (German short version) [43, 44] served as a measure for depressive symptoms to exclude participants with severe depression. A test of vocabulary (German: Wortschatztest) [45] was used to estimate the premorbid (crystallized) intelligence level.

**Fig. 3:**
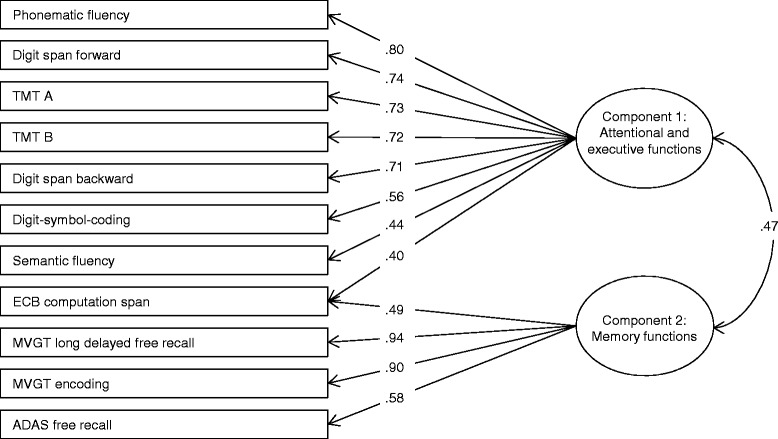
Results of the principal component analysis of cognitive measures. Two components were extracted, representing attention / executive functions (component 1) and memory (component 2). All weightings of at least *a*_ij_ = .40 are depicted. TMT A – Trail Making Test part A, TMT B – Trail Making Test part B, ECB – Everyday Cognition Battery, MVGT – Munich verbal memory test (adaptation of the California Verbal Memory Test), ADAS – Alzheimer’s Diseases Assessment Scale

To assess latent cognitive function scores, a principal component analysis was performed (see Additional file 1). In short, two components were extracted, one representing memory, the other representing attention / executive functions. Variables were *z*-standardized using means and standard deviations of the pre-test data. The two component scores represent the weighted average of those standardized variables with loadings of at least *a*_ij_ = .40 on the respective component (see Fig. 3). In addition, a global cognition score was built as the average of the two component scores and was used as the primary outcome^3^.

### Additional measures

At post-test, feasibility of the training programs was assessed with a short, self-constructed questionnaire. This questionnaire included an item on enjoyment and motivation associated with the training programs, in which the experienced enjoyment and motivation was rated on a 5-point rating scale.

### Statistical analyses

R version 3.1.2 [46] was used for statistical analyses. Baseline group differences were evaluated with one-way analyses of variance and *χ*^2^-tests for continuous and categorical variables, respectively. Correlations were calculated as Pearson product-moment correlations.

To investigate effects of the training interventions on cognition and associations of lifestyle with cognitive change, linear mixed effect models were conducted, using the nlme package 3.1.119 [47] in R. Global cognition was modelled with Group (contrasts CT vs. WLC and PT vs. WLC) × Time (pre vs. post) + Lifestyle (continuous) × Time as fixed effects in the same model and Subject as random intercept. Effects of training on cognition were indicated by significant Group × Time interactions, while associations of lifestyle with cognitive change over time were indicated by significant Lifestyle × Time interactions. The second aim was to compare the strength of association between cognitive change and training on the one hand and between cognitive change and lifestyle on the other hand. We therefore performed paired *t*-tests to compare the non-standardized *b*-coefficients of the Lifestyle × Time interaction with the ones of the contrasts (CT group vs. WLC group) × Time and (PT group vs. WLC group) × Time in the models.

For all models the normality distribution of the model residuals was assessed with quantile-quantile plots of the residuals and Shapiro-Wilk normality tests. The power to find small effects (*f* = 0.10) in the linear mixed effects models with α = 0.05 was calculated for the sample size of *N* = 54 with three groups and two time-points. Due to a high retest reliability of the cognitive composite scores (*r* ≥ .90, see Additional file 1), the calculated power was high (1 – β = .82).

Further exploratory analyses can be found in the Additional file 1: First, the stability of significant training effects was evaluated by the inclusion of the follow-up as a third time-point into the analysis. In addition, per protocol analyses were performed for the main outcomes (global cognition and composite scores), including only participants who completed at least 75 % of the training sessions and WLC participants (*n* = 48), to account for potential influences of training adherence. Furthermore, the Lifestyle × Time interaction was also evaluated for the three activity domain scores for variety of physical, of cognitive, and of social activities as lifestyle measures. Last, improvements in the CT program were analyzed with paired *t*-tests within the respective training group and correlations between change in training task performance and change in cognition were calculated. For improvements within the training program Cohen’s *d* was calculated as measure of effect size.

## Results

### Training time intervals and attendance

The training sessions or waiting period started 1 to 4 weeks after the pre-test (CT: *M* = 15.1 days, *SD* = 17.6 days, PT: *M* = 14.6 days, *SD* = 20.5 days) and lasted 10 weeks. One to four weeks after the last training session (CT: *M* = 5.0 days, *SD* = 8.4 days, PT: *M* = 13.2 days, *SD* = 10.2 days) the post-test was arranged. Time intervals between pre- and post-tests were similar in the WLC group (*M* = 16.1 weeks, *SD* = 5.6 weeks). The follow-up assessment was carried out after another 3 months (CT: *M* = 10.0 weeks, *SD* = 2.7 weeks, PT: *M* = 11.3 weeks, *SD* = 4.7 weeks, WLC: *M* = 15.7 weeks, *SD* = 13.5 weeks).

The participants in the cognitive training completed on average 49.89 sessions (*SD* = 7.56, range 25–55) of training. In the physical training group, the participants attended on average 15.41 group sessions (*SD* = 2.65, range 9–20). Most participants of the two training groups rated the training interventions as good or very good with regard to enjoyment and motivation (70 %, *n* = 19). Harms or unintended effects were not observed.

### Training- and lifestyle-related changes in cognition

**Fig. 4:**
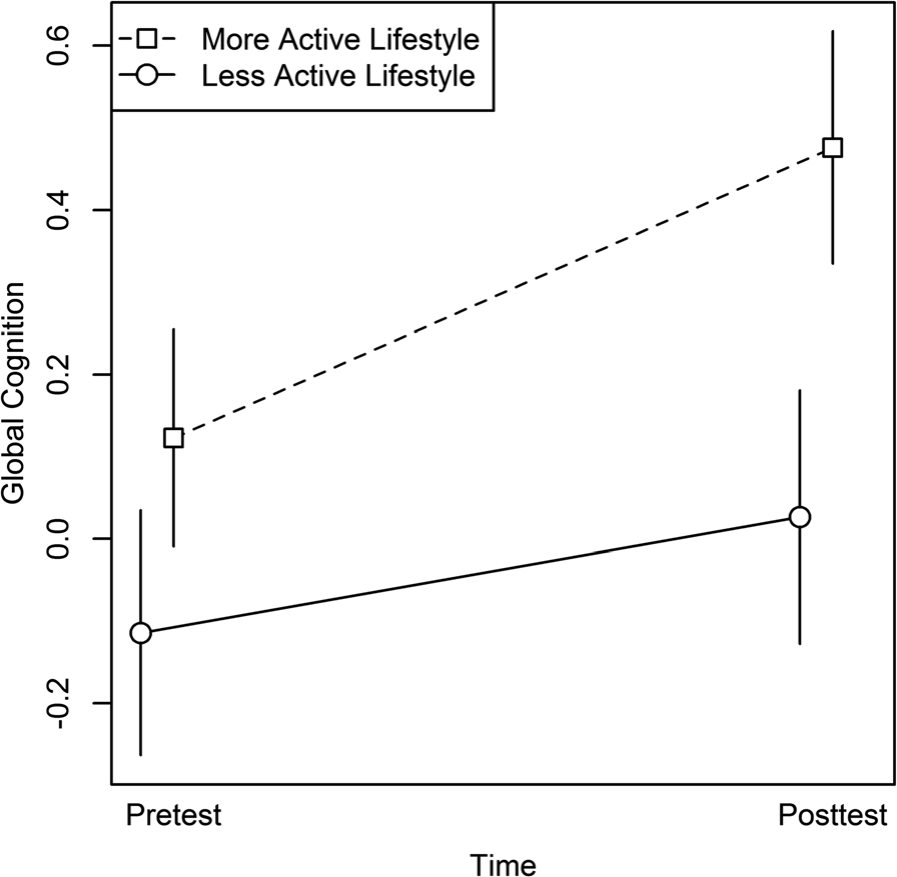
Global cognition as a function of lifestyle and time. Lifestyle was measured as variety of reported activities. For illustration purposes, the global cognition scores are depicted for individuals with a more active lifestyle (i.e., activity variety above median) versus individuals with a less active lifestyle (i.e., activity variety below median), at pre- and post-test. The median activity variety was 0.30. Error bars represent standard errors of the mean

There were significant main effects of Time, *F*(1,48) = 56.33, *p* < .001, and of Lifestyle, *F*(1,48) = 6.07, *p* = .02, on global cognition. Furthermore, the Lifestyle × Time interaction was significant, *F*(1,48) = 18.77, *p* < .001 (see Fig. 4), while the Group × Time interaction did not reach significance, *F*(2,48) = 2.64, *p* = .08.

**Fig. 5:**
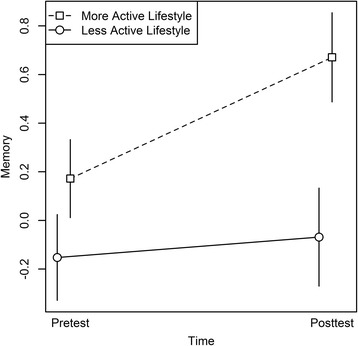
Memory as a function of lifestyle and time. Lifestyle was measured as variety of reported activities. The memory composite scores are depicted for individuals with a more active lifestyle (i.e., activity variety above median) versus individuals with a less active lifestyle (i.e., activity variety below median), at pre- and post-test. The median activity variety was 0.30. Error bars represent standard errors of the mean

The same pattern arose when modeling memory, with significant main effects of Time, *F*(1, 48) = 28.18, *p* < .001, and of Lifestyle, *F*(1,48) = 5.32, *p* = .03, as well as a significant Lifestyle × Time interaction, *F*(1,48) = 23.88, *p* < .001 (see Fig. 5). For modeling attention / executive functions, only the main effects of Time, *F*(1,48) = 19.28, *p* < .001, and of Lifestyle, *F*(1,48) = 4.57, *p* = .04, were significant, but no interaction effects.

Accounting for age, education, and cognitive status (MMSE) did not alter the results. For interaction effects on single cognitive test outcomes see Table 3.

**Table 3:** Training- and lifestyle-related changes in cognition from pre- to post-test

	Difference Post-Pre [95 % CI]			Group × Time		Lifestyle × Time	
Outcome measure	CT (*n* = 16)	PT (*n* = 18)	WLC (*n* = 20)	*F* statistic	*p*	*F* statistic	*p*
Global cognition	0.20 [0.03–0.37]	0.16 [0.01–0.30]	0.32 [0.22–0.43]	*F*(2,48) = 2.64	0.08	*F*(1,48) = 18.77	<0.001
Memory	0.34 [0.11–0.57]	0.15 [-0.10–0.40]	0.38 [0.19–0.58]	*F*(2,48) = 1.78	0.18	*F*(1,48) = 23.88	<0.001
Attention / executive functions	0.06 [-0.20–0.31]	0.16 [-0.03–0.36]	0.27 [0.11–0.42]	*F*(2,48) = 0.66	0.52	*F*(1,48) = 0.07	0.79
ADAS free recall	-0.34 [-0.89–0.22]	0.15 [-0.36–0.66]	-0.08 [-0.61–0.45]	*F*(2,48) = 1.33	0.27	*F*(1,48) = 2.47	0.12
ADAS recognition	0.00 [-1.13–1.13]	-0.44 [-1.27–0.38]	0.25 [-0.55–1.05]	*F*(2,47) = 0.71	0.50	*F*(1,47) = 1.06	0.31
ADAS orientation	0.00 [-0.48–0.48]	0.22 [-0.14–0.59]	0.10 [-0.11–0.31]	*F*(2,48) = 0.42	0.66	*F*(1,48) = 0.00	0.96
ADAS imagination	-0.19 [-0.48–0.1]	0.17 [-0.43–0.76]	-0.15 [-0.32–0.02]	*F*(2,48) = 0.93	0.40	*F*(1,48) = 0.38	0.54
ADAS naming	-0.12 [-0.55–0.3]	0.00 [-0.17–0.17]	0.00 [0.00–0.00]	*F*(2,48) = 0.43	0.65	*F*(1,48) = 0.08	0.78
ADAS verbal expression	0.00 [0.00–0.00]	0.00 [0.00–0.00]	0.00 [0.00–0.00]				
ADAS verbal comprehension	-0.06 [-0.20–0.07]	0.06 [-0.06–0.17]	-0.05 [-0.29–0.19]	*F*(2,48) = 0.44	0.65	*F*(1,48) = 0.80	0.38
ADAS word finding disturbances	-0.19 [-0.40–0.03]	-0.11 [-0.27–0.05]	-0.10 [-0.31–0.11]	*F*(2,48) = 0.05	0.95	*F*(1,48) = 0.00	0.99
CERAD figure copy	0.27 [-0.18–0.71]	0.33 [-0.23–0.90]	0.15 [-0.29–0.59]	*F*(2,47) = 0.47	0.63	*F*(1,47) = 0.02	0.89
CERAD figure recall	-1.14 [-2.68–0.39]	0.00 [-0.84–0.84]	-0.15 [-0.91–0.61]	*F*(2,46) = 0.90	0.41	*F*(1,46) = 0.85	0.36
CERAD Boston Naming Test	0.06 [-0.47–0.59]	-0.17 [-1.78–1.44]	0.20 [-0.25–0.65]	*F*(2,48) = 0.16	0.85	*F*(1,48) = 0.17	0.68
TMT A	0.36 [0.02–0.71]	0.22 [-0.14–0.59]	0.51 [0.12–0.91]	*F*(2,48) = 0.73	0.49	*F*(1,48) = 2.07	0.16
TMT B	-0.01 [-0.46–0.43]	0.28 [-0.14–0.70]	0.20 [-0.03–0.43]	*F*(2,48) = 0.22	0.81	*F*(1,48) = 1.71	0.20
Phonematic fluency	0.06 [-0.31–0.43]	0.48 [-0.11–1.07]	0.45 [-0.003–0.91]	*F*(2,48) = 0.79	0.46	*F*(1,48) = 1.64	0.21
Semantic fluency	0.20 [-0.10–0.50]	-0.02 [-0.29–0.26]	0.23 [-0.12–0.57]	*F*(2,48) = 0.70	0.50	*F*(1,48) = 0.11	0.74
MVGT encoding	0.47 [0.16–0.78]	0.34 [-0.04–0.71]	0.66 [0.37–0.95]	*F*(2,47) = 1.46	0.24	*F*(1,47) = 15.96	<0.001
MVGT delayed free recall	0.68 [0.39–0.97]	-0.00 [-0.41–0.41]	0.49 [0.27–0.72]	*F*(2,45) = 6.62	0.003	*F*(1,45) = 9.91	0.003
MVGT recognition	1.27 [-0.26–2.80]	0.78 [-0.03–1.59]	-0.28 [-1.02–0.46]	*F*(2,46) =2.35	0.11	*F*(1,46) = 0.14	0.71
Digit span forward	-0.03 [-0.56–0.50]	-0.19 [-0.71–0.33]	0.07 [-0.33–0.47]	*F*(2,48) = 0.58	0.57	*F*(1,48) = 0.23	0.64
Digit span backward	-0.28 [-0.86–0.30]	0.31 [-0.19–0.81]	0.14 [-0.29–0.57]	*F*(2,48) = 0.73	0.49	*F*(1,48) = 0.79	0.38
Digit-symbol-coding	0.12 [-0.35–0.59]	-0.11 [-0.35–0.14]	0.19 [-0.10–0.48]	*F*(2,48) = 1.83	0.17	*F*(1,48) = 0.09	0.76
ECB computation span	0.22 [-0.20–0.65]	0.19 [-022–0.59]	0.33 [-0.03–0.69]	*F*(2,44) = 0.26	0.77	*F*(1,44) = 2.00	0.16

Depicted are the mean differences in cognitive measures between pre- and post-test within the three groups and 95 % confidence intervals in brackets, as well as statistics for Group × Time and Lifestyle × Time interactions

*CT* Cognitive training group, *PT* Physical training group, *WLC* Wait-list control group. *ADAS*, Alzheimer’s Diseases Assessment Scale, *CERAD* Consortium to Establish a Registry for Alzheimer’s Disease, *TMT* Trail Making Test (part A and B), *MVGT* German adaptation of the California Verbal Learning Test, *ECB* Everyday Cognition Battery

### Comparison of lifestyle and training associations

The Lifestyle × Time interaction, *b* = 1.40, was significantly larger than the one of (CT vs. WLC) × Time, *b* = -0.05, *t*(48) = 4.50, *p* < .001, or the one of (PT vs. WLC) × Time, *b* = -0.13, *t*(48) = 4.74, *p* < .001, in the model of global cognition. Likewise, the Lifestyle × Time interaction, *b* = 2.68, was significantly larger than the one of (CT vs. WLC) × Time, *b* = 0.02, *t*(48) = 4.89, *p* < .001, or the one of (PT vs. WLC) × Time, *b* = -0.17, *t*(48) = 5.18, *p* < .001, in modeling the memory composite score. There was no significant difference between the *b*-coefficient of the Lifestyle × Time interaction, *b* = 0.13, and the ones of (CT vs. WLC) × Time, *b* = -0.11, *t*(48) = 0.50, *p* = .31, or (PT vs. WLC) × Time, *b* = -0.10, *t*(48) = 0.48, *p* = .32, in the model of attention / executive functions.

## Discussion

We investigated effects of cognitive and physical training on cognition, in the context of lifestyle-related changes in cognition. In addition, we compared the strength of association between cognitive change and training on the one hand, and between cognitive change and lifestyle on the other hand. Neither the PT nor the CT group improved in global cognition after 10 weeks of training compared to the WLC condition. In contrast, the self-reported lifestyle, defined as the variety of regular leisure activities (i.e., the number of different activities) in a typical week before study participation, was associated with changes in global cognition over the same period. Individuals with a more active lifestyle demonstrated a favorable change in cognitive performance during the study period compared to individuals with a less active lifestyle. This association was irrespective of the intervention group to which the participants had been assigned. Moreover, the association of lifestyle with cognitive change was significantly stronger than the association of training with cognitive change. Accounting for cognitive status, age, and education did not affect the lifestyle associations, implicating that influences of these covariates on the lifestyle-related changes in cognition are unlikely.

Unexpectedly, we did not observe any cognitive benefits of the cognitive and physical training programs. Previous research has also produced mixed results regarding training outcomes [16, 20]. Several factors may influence effects of training on cognition, such as the nature of the training programs. We applied a multimodal physical training program in this study. The great majority of physical training effects are based on aerobic training, though a small but increasing number of resistance training experiments have also shown promising effects [48, 49]. Another factor may constitute the investigated sample of older adults at risk of dementia. Training may be less effective in these at risk populations [16, 34] than in healthy older adults [31, 50]. Finally, the cognitive training program with an emphasis on auditory processing might not have recruited the assessed cognitive outcomes. However, high correlations between CT performance and global cognition at pre-test do not support this assumption (see Additional file 1). Rather, the lack of an association between improvement in the cognitive training tasks and improvements in global cognition indicates that the transfer from training gains to global cognitive domains was low. That is, although there were improvements within the cognitive training tasks, these did not generalize to global cognitive benefits.

The observed relationship between an active lifestyle and cognitive change in this study is in line with prospective studies demonstrating a substantial risk reduction of cognitive decline and dementia in individuals with higher physical [3] or cognitive [4, 51] activity and in particular in individuals with a higher *variety* of physical and cognitive activities [5–7]. The study extends previous work in that it revealed that the associations of lifestyle with cognitive change were stronger than the effects yielded with specifically designed training programs in older adults at risk of dementia. Physical, cognitive and social activity are main protective lifestyle factors against cognitive decline and dementia. We thus operationalized lifestyle by the amount of activity, in which the participants usually engage. In order to evaluate training effects in the context of lifestyle activity, we assessed lifestyle in all participants at the beginning of the study. Another interesting option to directly compare lifestyle and training effects would be to design an “active lifestyle intervention”, in which previously sedentary adults engage into different, unspecific leisure activities, and compare its effect to the ones of a specific training intervention (similar to a study of Stine-Morrow and colleagues [52]).

The association of lifestyle with change in cognitive performance was only observed for memory, but not for attention and executive functions. Similarly, Park and colleagues [53] reported specific effects of engagement in novel tasks on memory, but not on other cognitive domains. The finding is also in line with a large number of animal studies demonstrating benefits in learning and memory of animals placed in an “enriched environment”, i.e. a condition which enables cognitive, physical, and social activity [54–56]. Effects on hippocampal volume and memory have also been associated with physical [57] as well as cognitive activity [58] in humans. Meta-analyses on physical exercise reported effects in particular on executive functions [18], but also on memory [20]. The specific relationship of lifestyle with memory, but not with attentional and executive functions, implies that different mechanisms may underlie and influence the course of both domains.

Variety of activities within all three activity subdomains (cognitive, physical, social activities) was significantly associated with changes in global cognition and in memory, indicating that it is not one specific activity domain which is most favorable (see Additional file 1).

If an active lifestyle causes beneficial effects on cognition indeed, then the question arises why specifically designed physical and cognitive training programs fail to produce corresponding results. There are several aspects in which activities of an active lifestyle and training interventions may differ: First, the intrinsic motivation and experienced enjoyment may be different between training tasks and leisure activities. The desire to engage in activities is predictive for activity-induced structural brain changes, indicating that motivation plays an important role in affecting cognitive change [59]. However, most participants in this study found the training interventions motivating and enjoyable. Thus, it seems unlikely that the absence of training effects on cognition was due to a lack of enjoyment or motivation. Second, leisure activities and training interventions may differ with respect to activity dosage and duration: Leisure activities might have been pursued more frequently or for a longer period of time. And third, the training interventions consisted of specific, but only few activity types, namely six working-memory and auditory-discrimination tasks in the cognitive training program and endurance, coordination, balance, flexibility, and strengthening components in the physical training program. In contrast, the assessed lifestyle of the participants comprised three to 14 different socially, cognitively, or physically demanding activities, each involving many different tasks. Variation of tasks might be a crucial factor in inducing generalizing effects on global cognition [5–7, 60] and may be more effective than repeated training of a limited number of tasks [26]. In line with this notion, Angevaren and colleagues [5] demonstrated that cognitive function was associated with the number of different physical leisure activities, but not with the time spent with physical exercise per week. Finally, an active lifestyle comprises activities of different domains such as physical and cognitive activities, which may have synergistic effects on cognition [27].

This study has several limitations: The variety of activities, as our measure of lifestyle, was only observed and not experimentally manipulated. Hence, a causal effect of lifestyle on cognitive change cannot be inferred. To exclude reverse causality, that is, an effect of cognitive status on lifestyle, we statistically accounted for cognitive status. This did not alter the significant associations of lifestyle with cognitive change. As mentioned above, our sample size of 54 participants constrained power to detect effects. However, due to the high measurement accuracy resulting from the extensive cognitive test battery, the sample size was sufficient to detect small effects with a high power. The small sample size might be a reason for the lack of significant training effects, but is not an explanation for stronger associations of lifestyle than of training with cognitive change. Last, the outcome of a training intervention on cognition may be moderated by the previous fitness or activity level [61]. As in the present study the sample was not restricted to sedentary older adults, the relatively moderate activity level of the participants might have reduced effects of the training interventions.

Further research is needed in order to establish recommendations for patients. The assessment of lifestyle variables should be considered in future interventional training studies to investigate the impact of lifestyle on the efficacy of training programs (moderating effect). The present study provides a first indication, that lifestyle factors might have a stronger impact on cognition than training programs. It is thus important to investigate whether a change towards a more active lifestyle in general, with multiple cognitive, physical, and social activities, is effective and more advantageous than the engagement in specific training programs. Furthermore, the mentioned key factors which may be critical for the positive associations of an active lifestyle (such as duration, frequency, variety, multimodality, motivation, and enjoyment of activities) should be pursued in order to design more efficient training programs.

## Conclusions

Lifestyle activity but not specific training interventions were associated with changes in cognition. These results demonstrate that an active lifestyle must contain further factors (besides physical and cognitive exercise) which may play a role for effects on cognition. Further experimental studies are necessary to investigate these factors which may account for the beneficial effects of an active lifestyle, such as variety, dosage or experienced enjoyment. Incorporating these factors in newly designed programs may then results in more efficient interventions than currently available cognitive and physical training programs.

## Additional file

Additional file 1: Additional methods and results. The document contains more detailed information of methods regarding the principal component analysis to retrieve cognitive component scores, and the generation of the lifestyle activity scores. It further describes additional results, including results of the 3-month follow-up and associations of cognitive training tasks with the assessed cognitive outcomes. (DOCX 42 kb)

## Abbreviations

CT: Cognitive training
MMSE: Mini-Mental State Examination
PT: Physical training
WLC: Wait-list control

## Footnotes

1 Initial exclusion criterion was changed from MMSE < 22 to MMSE < 20, in order to allow the participation of participants with probable mild dementia and the range for mild dementia usually includes MMSE scores of 21 and 20.

2 The number of participants was reduced from initially planned 100, as the retest reliability of the primary outcome was higher than expected, which resulted in a high power to detect small effects already in 65 included participants.

3 We refrained from the ADAS-Cog sum-score as the previously defined primary outcome and used a global composite score instead, as its skewed distribution indicated that ADAS-Cog was prone to ceiling effects in the applied cohort. Besides, composite scores of cognition reduce alpha-error inflation which results from multiple testing and became the gold standard in recent years in interventional trials that assess cognitive change as the primary outcome.
