# Supplementary material for: Cognitive change is more positively associated with an active lifestyle than with training interventions in older adults at risk of dementia: a controlled interventional clinical trial
Source: BMC Psychiatry. 2016 Sep 8;16(1):315. doi: 10.1186/s12888-016-1018-z (PMC5016950; doi:10.1186/s12888-016-1018-z)
Supplement: Additional file 1: — Additional methods and results. The document contains more detailed information of methods regarding the principal component analysis to retrieve cognitive component scores, and the generation of the lifestyle activity scores. It further describes additional results, including results of the 3-month follow-up and associations of cognitive training tasks with the assessed cognitive outcomes. (DOCX 42 kb) [file 12888_2016_1018_MOESM1_ESM.docx]

Additional methods

Principal component analysis of cognitive measures

To assess latent cognitive function scores, a principal component analysis was performed including all screened participants with complete cognitive baseline tests (*n* = 64). An oblique rotation technique (OBLIMIN) was chosen, as correlations between the extracted cognitive components were assumed. The Kaiser criterion (eigenvalues ≥ 1.0) was used to determine the number of extracted components. Eleven cognitive variables were included in the principal component analysis: Munich verbal memory test (MVGT) encoding, MVGT long delayed free recall, free recall of the Alzheimer’s Disease Assessment Scale, working memory in the Everyday Cognition Battery, Trail Making Test A and B, digit span forward and backward, digit-symbol-coding and semantic and phonematic fluency. Not included into the principal component analysis were figure copy and recall and the Boston Naming Test of the Consortium to Establish a Registry for Alzheimer’s Disease test battery, the subtests recognition, orientation, imagination, naming, verbal expression, verbal comprehension, and word finding disturbances of the Alzheimer’s Disease Assessment Scale, as well as MVGT recognition, as 30% of the participants or more had achieved the best or second best score on these scales at baseline, precluding improvement on these scales. Two components were extracted, one representing memory with high loadings of working memory and episodic memory scores, the other component representing attention / executive functions with high loadings of processing speed, task switching, and verbal fluency (see Figure 3 in the main article).

All variables were *z*-standardized by using the pre-test data of the analyzed training sample (*n* = 54) and two component scores were built representing the weighted average of those *z*-standardized variables with loadings of at least *a*_ij_ = .40 on the respective component. In addition, a global cognition score was built as the average of the two component scores. In the case of missing variables, the respective variables were excluded for the subject at all time-points and the component scores were calculated with the remaining variables, if less than half of the variables of a component score were missing. In the case that more variables were missing at a time point, the respective component score and the global cognition score were not built for this individual and time point. Retest reliability, assessed in the wait-list control group, was very good for global cognition as the primary outcome (pre – post, *r* = .96, and pre – follow-up, *r* = .97), and both secondary outcomes, memory (pre – post, *r* = .93, and pre – follow-up, *r* = .96) and attention / executive functions (pre – post, *r* = .90, and pre – follow-up, *r* = .84).

Rating of lifestyle activity domains

With the Community Healthy Activities Model Program for Seniors Physical Activity Questionnaire for Older Adults [1] the frequency and duration of 40 different physical, cognitive, and social activities were assessed. We categorized the activities to three activity domains (physical, cognitive, and social) on the basis of independent ratings of three authors (PF, OCK, DL). Each activity was rated with respect to cognitive and physical demands as well as to the amount of social interaction on a five-point rating scale from 1 (*no demands*) to 5 (*high demands*). All activities with a mean author rating over 3 (*moderate demands*) were categorized to the respective domain (see Table A1). The ratings were validated with ratings of 39 cognitively healthy older adults (Mini-mental state examination ≥ 26, aged 64-90, not participants of the present study). The correlations between the authors’ ratings and the seniors’ ratings were very high for all domains (physical domain: *r* = .87, cognitive domain: *r* = .89, social domain: *r* = .84). We adapted the categorization to better fit the seniors’ opinion, if their ratings clearly indicated the inclusion (values > 3.5) or the exclusion (values < 2.5) of the activity to the particular domain. By this procedure ”Yoga or Tai Chi” was additionally included in the cognitive domain, ”golf (with wearing equipment)”, “singles tennis”, and “aerobic” were additionally included in the social domain, and “golf (with wearing equipment)” was additionally included in the physical domain. Twelve activities with low physical, cognitive, and social demands were not categorized to any domain. The reliability of ratings was very high. Authors’ ratings revealed a very good Cronbach’s α for all domains (physical domain: α_Cronbach_ = .92, cognitive domain: α_Cronbach_ = .86, social domain: α_Cronbach_ = .95). Three domain scores for physical, cognitive, and social activities as well as one overall activities score were built. Each domain score reflects the percentage of performed, domain-specific activities in relation to the possible number of activities in this domain. The overall activities score was built by averaging the three domain scores.

Additional results

Training- and lifestyle-related changes in cognition from pre-test to follow-up

Similarly to the main analysis of training effects and associations of lifestyle with cognitive change between pre- and post-test, linear mixed effect models were conducted to analyze associations with change in cognition in the time between pre-test and follow-up. Global cognition as well as memory and attention / executive functions were modelled with Time (pre, post, and follow-up) × Group (cognitive training [CT], physical training [PT], wait-list control [WLC]) + Time × Lifestyle as fixed effects and Subject as random intercept.

There were significant main effects of time, *F*(2,77) = 39.19, *p* < .001, and of lifestyle, *F*(1,48) = 6.21 , *p* = .02, on global cognition. Furthermore, the Lifestyle × Time interaction was significant, *F*(2,77) = 8.12, *p* = .001, while the Group × Time interaction was not significant, *F*(4,77) = 1.92, *p* = .12.

There were also significant main effects of time, *F*(2,77) = 31.16, *p* < .001, and of lifestyle, *F*(1,48) = 5.68, *p* = .02, and a significant Lifestyle × Time interaction, *F*(2,77) = 12.51, *p* < .001, when modeling memory, but no Group × Time interaction, *F*(4,77) = 1.11, *p* = .36. For modeling attention / executive functions, only the main effects of time, *F*(2,76) = 10.32, *p* < .001, and of lifestyle, *F*(1,48) = 4.58, *p* = .04, were significant, but no interaction effects, *p*s > .36.

Per protocol analyses

Per protocol analyses were performed for global cognition and the composite scores memory and attention / executive functions, as the main outcomes. These analyses including only participants who completed at least 75% of the training sessions and WLC participants (*n* = 48), to account for potential influences of training adherence. Per protocol analyses did not alter the results regarding Group × Time and Lifestyle × Time interactions on cognition.

Associations of activity subdomains with cognitive change over time

Regarding the three activity domains, all three domain scores had significant Lifestyle × Time interactions on global cognition, *p*s < .008, and on memory, *p*s < .002, but not on attention / executive functions, *p*s > .73.

Improvements within the training programs

Within the CT group, global cognition and the memory composite score at pre-test were associated with performance in the training tasks “tell us apart” and “sound replay”, but not with performance in “high or low” or “match it” at the beginning of the training period (third session). “Sound replay” was also associated with the composite score of attention/ executive functions. At post-test, the final training performances in “tell us apart”, “match it”, and “sound replay” were associated with the global cognition post-test score (for further information see Table A2). Within the CT group, the performance increased from beginning to end of training in the trained tasks “high or low”, *t*(12) = -5.27, *p* < .001, Cohen’s *d* = 1.46, “tell us apart”, *t*(13) = 4.71, *p* < .001, Cohen’s *d* = 1.26, and “match it”, *t*(13) = 3.77, *p* = .002, Cohen’s *d* = 1.01, but not in the task “sound replay”, *t*(13) = 1.42, *p* = .18, Cohen’s *d* = 0.38. However, improvements in the trained tasks were not significantly associated with improvements in global cognition, memory, or attention / executive functions (*p*s > .25, see Table A2).

List of abbreviations used

CT: cognitive training; MVGT: Munich verbal memory test (adaptation of the California Verbal Memory Test); PT: physical training; WLC: wait-list control.

References

1. Stewart AL, Mills KM, King AC, Haskell WL, Gillis D, Ritter PL. CHAMPS physical activity questionnaire for older adults: outcomes for interventions. Med Sci Sports Exerc. 2001;33(7):1126-41. doi:10.1097/00005768-200107000-00010.

Tables

**Table A1. Categorization of activities into social, physical, and cognitive domains.**

| Activity | Rating scores | | | % Subjects |
| --- | --- | --- | --- | --- |
|  | Cognitive domain | Social domain | Physical domain |  |
| **Multidomain activities^a^** |  |  |  |  |
| Play basketball, soccer or racquetball | 3.7 | 4.3 | 5.0 | 10 |
| Play singles tennis | 3.3 | 3.7^d^ | 5.0 | 6 |
| Play doubles tennis | 3.3 | 3.3 | 5.0 | 0 |
| Dance | 3.3 | 4.3 | 4.0 | 13 |
| Play cards and board games | 4.3 | 4.7 |  | 29 |
| Visit family or friends | 3.3 | 5.0 |  | 81 |
| Do volunteer work | 4.0 | 4.0 |  | 56 |
| Attend club meetings | 3.3 | 4.7 |  | 50 |
| Attend cultural events | 4.0 | 3.3 |  | 62 |
| Do Yoga or Tai Chi | 3.5^d^ |  | 3.3 | 4 |
| Do aerobic |  | 3.5^d^ | 4.7 | 0 |
| Play golf, with carrying equipment |  | 3.6^d^ | 3.8^d^ | 0 |
| **Single domain activities^b^** |  |  |  |  |
| Play musical instruments | 5.0 |  |  | 10 |
| Use a computer | 4.3 |  |  | 73 |
| Read | 3.7 |  |  | 98 |
| Do arts and crafts | 3.3 |  |  | 38 |
| Go to the senior center |  | 4.3 |  | 25 |
| Attend church activities |  | 3.3 |  | 38 |
| Jog or run |  |  | 4.7 | 21 |
| Swim moderately or fast |  |  | 4.7 | 13 |
| Skate (ice, roller, in-line) |  |  | 4.0 | 2 |
| Use an aerobic machine |  |  | 4.3 | 19 |
| Do moderate/heavy strength training |  |  | 4.3 | 15 |
| Walk uphill or hike |  |  | 3.7 | 46 |
| Do heavy gardening |  |  | 3.7 | 35 |
| Do water exercise |  |  | 3.7 | 19 |
| Bicycle |  |  | 3.7 | 71 |
| Do heavy work around the house |  |  | 3.3 | 50 |
| **Low demand activities^c^** |  |  |  |  |
| Play golf, riding in a cart |  |  |  | 0 |
| Shot pool or billiards |  |  |  | 2 |
| Do light work around the house |  |  |  | 88 |
| Do light gardening |  |  |  | 50 |
| Work on machinery |  |  |  | 23 |
| Walk fast or briskly |  |  |  | 33 |
| Walk to do errands |  |  |  | 71 |
| Walk leisurely |  |  |  | 62 |
| Swim gently |  |  |  | 21 |
| Do stretching or flexibility |  |  |  | 58 |
| Do light strength training |  |  |  | 23 |
| General conditioning exercises |  |  |  | 25 |

*Note*. Depicted are mean ratings on a five-point rating scale from 1 (no demands) to 5 (high demands), for ratings higher than 3. % Subjects = Percentage of subjects who had engaged into the respective activity.  ^a^Two and three domains with rating > 3. ^b^One domain with rating > 3. ^c^No domain with rating > 3. ^d^Categorization adapted to senior ratings.

**Table A2. Associations of training task performance with cognitive outcomes in the cognitive training group.**

|  | Global cognition pre | | Memory pre | | Attention / executive functions pre | | |
| --- | --- | --- | --- | --- | --- | --- | --- |
|  | *r* | *p* | *r* | *p* | *r* | | *p* |
| Start high or low^a^ | -.07 | .83 | .02 | .95 | -.17 | | .57 |
| Start tell us apart | .58 | .03 | .63 | .02 | .44 | | .12 |
| Start match it | -.13 | .65 | -.23 | .42 | .02 | | .94 |
| Start sound replay | .62 | .02 | .57 | .03 | .62 | | .02 |
|  | Global cognition post | | Memory post | | Attention / executive functions post | | |
|  | *r* | *p* | *r* | *p* | *r* | *p* | |
| End high or low^a^ | -.46 | .11 | -.51 | .07 | -.23 | .45 | |
| End tell us apart | .59 | .03 | .65 | .01 | .33 | .24 | |
| End match it | .76 | .002 | .66 | .01 | .73 | .003 | |
| End sound replay | .54 | .046 | .35 | .22 | .72 | .003 | |
|  | Difference global cognition | | Difference memory | | Difference attention / executive functions | | |
|  | *r* | *p* | *r* | *p* | *r* | *p* | |
| Difference high or low^a^ | -.28 | .36 | -.39 | .19 | -.03 | .93 | |
| Difference tell us apart | -.03 | .91 | .35 | .21 | -.37 | .19 | |
| Difference match it | -.13 | .67 | -.15 | .61 | -.03 | .92 | |
| Difference sound replay | .33 | .25 | .23 | .42 | .23 | .43 | |

*Note*. Data of 14 cognitive training participants. Start = training task performance at the beginning of the training period (third session), End = training task performance at the end of the training period (last session), Difference = difference in performance between beginning and end of the training program and between pre- and post-test of the cognitive outcomes, respectively. ^a^Lower scores indicate better performance.
